# Supplementary material for: A multi-objective mathematical model of a water management problem with environmental impacts: An application in an irrigation project
Source: PLoS One. 2021 Aug 3;16(8):e0255441. doi: 10.1371/journal.pone.0255441 (PMC8330924; doi:10.1371/journal.pone.0255441)
Supplement: S1 Table — This includes results that we have found from using the Non-dominated Sorting Genetic Algorithm-II (NSGA-II) on the Multi-objective Optimisation Problem (MOP). (PDF) [file pone.0255441.s001.pdf]

S1 Table. Details of 1-17 Pareto solutions for the crops.

Solutions	Land area for each crop (ha)										NR $\times 10^7$ AUD	EFD (GL)
	T. Aus	T. Aman	Boro Rice	Wheat	Potato	Oil Seeds	Pulses	Sugarcane	Winter Vegetable	Summer Vegetable		
1	1452.18	1516.63	13504.46	2555.28	48610.52	6567.29	1072.37	69228.00	69227.79	16982.25	1877.48	35.53
2	1452.20	1516.63	13504.54	2545.76	48659.10	6569.55	1072.40	69228.00	69227.79	16982.02	1877.47	29.52
3	1452.89	1515.99	13504.50	2542.22	48640.24	6570.11	1072.34	69228.00	69227.77	16981.96	1877.43	28.15
4	1452.83	1515.95	13504.20	2557.02	48639.17	6570.94	1072.33	69228.00	69227.78	16985.11	1877.43	28.10
5	1452.18	1516.23	13504.35	2553.43	48621.53	6569.99	1072.36	69228.00	69227.79	16984.37	1877.42	28.07
6	1451.27	1507.02	13506.34	2552.25	48677.75	6505.70	1072.36	69228.00	69227.60	16987.30	1877.41	23.48
7	1451.39	1507.87	13505.91	2561.68	48659.80	6507.62	1072.37	69228.00	69227.60	16982.33	1877.40	23.04
8	1451.34	1509.33	13506.18	2552.71	48677.18	6505.74	1072.38	69228.00	69227.60	16982.50	1877.39	20.20
9	1451.32	1507.90	13506.36	2549.53	48661.38	6505.73	1072.37	69228.00	69227.60	16982.80	1877.38	20.06
10	1451.49	1515.86	13506.96	2555.23	48642.92	6566.26	1072.38	69228.00	69227.78	16989.75	1877.32	19.73
11	1452.97	1517.59	13506.43	2520.32	48670.56	6539.19	1072.36	69228.00	69227.79	16984.37	1877.28	19.42
12	1452.33	1517.27	13506.43	2543.84	48682.23	6540.07	1072.35	69228.00	69227.79	16982.17	1877.27	16.11
13	1452.34	1517.28	13506.44	2543.94	48679.75	6539.20	1072.35	69228.00	69227.79	16982.35	1877.27	16.08
14	1442.61	1522.08	13499.21	2854.59	48290.12	6538.69	1071.55	69228.00	69227.78	17059.79	1877.13	07.17
15	1442.54	1521.12	13499.07	2861.68	48298.97	6537.46	1071.56	69228.00	69227.78	17060.56	1877.13	05.41
16	1442.55	1521.20	13499.25	2861.65	48301.76	6537.01	1071.55	69228.00	69227.78	17059.33	1877.13	04.93
17	1441.94	1522.22	13504.05	2847.55	48308.61	6535.15	1071.56	69228.00	69227.78	17058.75	1877.05	03.19
