# Supplementary material for: A multi-objective mathematical model of a water management problem with environmental impacts: An application in an irrigation project
Source: PLoS One. 2021 Aug 3;16(8):e0255441. doi: 10.1371/journal.pone.0255441 (PMC8330924; doi:10.1371/journal.pone.0255441)
Supplement: S2 Table — This includes results that we have found from using the Non-dominated Sorting Genetic Algorithm-II (NSGA-II) on the Multi-objective Optimisation Problem (MOP). (PDF) [file pone.0255441.s002.pdf]

S2 Table. Details of 18-34 Pareto solutions for the crops.

Solutions	Land area for each crop (ha)										NR $\times 10^7$ AUD	EFD (GL)
	T. Aus	T. Aman	Boro Rice	Wheat	Potato	Oil Seeds	Pulses	Sugarcane	Winter Vegetable	Summer Vegetable		
18	1443.71	1521.77	13504.04	2846.21	48306.15	6539.21	1071.59	69228.00	69227.78	17060.69	1877.02	2.72
19	1443.16	1522.15	13503.99	2856.10	48294.13	6538.03	1071.57	69228.00	69227.79	17060.71	1877.01	2.67
20	1443.33	1522.05	13504.04	2849.83	48298.26	6537.86	1071.52	69228.00	69227.79	17061.53	1876.99	2.66
21	1443.33	1522.05	13504.04	2849.83	48298.26	6537.86	1071.52	69228.00	69227.79	17061.53	1876.99	2.10
22	1443.50	1521.45	13504.08	2859.98	48296.24	6539.67	1071.58	69228.00	69227.78	17060.71	1876.99	0.80
23	1452.84	1527.87	13531.50	2864.28	48205.75	6538.35	1071.70	69227.99	69227.68	17036.93	1876.98	0.362
24	1452.90	1528.23	13531.42	2864.45	48207.59	6538.36	1071.70	69227.99	69227.68	17037.71	1876.98	0.36
25	1452.83	1525.98	13531.47	2864.31	48205.78	6538.96	1071.68	69227.99	69227.69	17036.79	1876.98	0.35
26	1448.81	1551.75	13521.38	2871.88	48181.63	6520.27	1071.81	69228.00	69227.78	17041.33	1876.95	0.04
27	1448.64	1550.46	13521.32	2851.32	48182.81	6522.58	1071.79	69228.00	69227.78	17045.05	1876.87	0.017
28	1448.88	1551.25	13520.29	2851.37	48184.63	6522.41	1071.79	69228.00	69227.78	17044.01	1876.87	0.0152
29	1448.64	1551.21	13519.73	2851.22	48187.65	6522.12	1071.79	69228.00	69227.78	17044.49	1876.87	0.0151
30	1449.05	1552.30	13521.16	2851.44	48180.74	6522.62	1071.79	69228.00	69227.78	17043.59	1876.87	0.0148
31	1448.76	1551.92	13521.71	2841.49	48186.76	6521.53	1071.79	69228.00	69227.92	17043.48	1876.87	0.0133
32	1448.93	1551.42	13519.54	2851.25	48185.32	6523.30	1071.77	69228.00	69227.78	17044.14	1876.85	4.5×10^{-5}
33	1448.77	1552.20	13519.19	2851.26	48185.06	6531.23	1071.77	69228.00	69227.78	17043.90	1876.85	3.4×10^{-5}
34	1453.53	1529.59	13451.38	2854.91	48197.63	6631.99	1072.15	69227.99	69227.88	17026.76	1876.68	0.00
