# Supplementary material for: A multi-objective mathematical model of a water management problem with environmental impacts: An application in an irrigation project
Source: PLoS One. 2021 Aug 3;16(8):e0255441. doi: 10.1371/journal.pone.0255441 (PMC8330924; doi:10.1371/journal.pone.0255441)
Supplement: S3 Table — This includes results that we have found from using the Non-dominated Sorting Genetic Algorithm-II (NSGA-II) on the Multi-objective Optimisation Problem (MOP). (PDF) [file pone.0255441.s003.pdf]

S3 Table. Details of 1-17 Pareto solutions for the environmental flow.

Solution	Jan	Feb	Mar	Apr	May	Jun	Jul	Aug	Sep	Oct	Nov	Dec
1	99.03	97.53	90.94	99.82	128.59	163.71	101.79	101.92	92.15	144.99	249.63	84.99
2	99.07	97.52	90.70	99.82	128.53	163.65	101.81	102.09	98.50	144.97	249.64	84.88
3	99.05	97.52	91.72	99.82	128.91	163.86	101.81	103.47	98.59	143.41	251.05	85.15
4	99.09	97.56	91.45	99.82	128.57	164.00	101.80	103.44	98.61	144.97	251.08	85.37
5	99.18	97.53	91.76	99.82	128.16	163.58	101.57	103.33	98.60	144.62	250.99	85.03
6	99.10	97.76	98.16	99.82	128.97	148.95	108.04	104.21	98.47	150.96	250.91	83.22
7	99.23	97.68	96.60	99.81	128.92	148.57	108.01	104.13	98.59	150.75	250.93	85.05
8	99.12	97.75	102.24	99.82	128.62	149.37	107.98	104.18	98.57	150.95	247.60	84.54
9	99.10	97.69	102.42	99.82	128.93	149.27	107.96	104.20	98.59	150.95	247.33	84.74
10	102.56	97.41	98.15	99.85	129.43	161.82	102.16	104.14	98.58	150.96	249.81	86.28
11	99.04	97.55	100.02	99.83	135.46	163.16	101.85	103.43	98.64	150.70	251.05	85.51
12	99.06	97.50	100.11	99.83	135.23	162.97	101.85	103.43	98.67	150.83	250.93	88.82
13	99.06	97.50	100.03	99.83	135.27	161.97	101.85	103.43	98.68	150.81	250.93	88.86
14	98.97	99.79	97.85	100.00	129.43	164.92	102.12	103.78	96.24	143.52	247.57	105.05
15	98.97	99.95	97.84	99.99	129.42	164.71	102.12	103.78	97.83	143.62	247.58	104.77
16	98.99	99.95	98.09	99.99	129.38	164.73	102.15	103.77	98.05	143.72	247.55	104.77
17	99.43	99.82	97.56	100.02	126.74	165.46	102.19	103.75	100.40	150.36	251.43	105.16
