# Supplementary material for: A multi-objective mathematical model of a water management problem with environmental impacts: An application in an irrigation project
Source: PLoS One. 2021 Aug 3;16(8):e0255441. doi: 10.1371/journal.pone.0255441 (PMC8330924; doi:10.1371/journal.pone.0255441)
Supplement: S4 Table — This includes results that we have found from using the Non-dominated Sorting Genetic Algorithm-II (NSGA-II) on the Multi-objective Optimisation Problem (MOP). (PDF) [file pone.0255441.s004.pdf]

S4 Table. Details of 18-34 Pareto solutions for the environmental flow.

Solution	Jan	Feb	Mar	Apr	May	Jun	Jul	Aug	Sep	Oct	Nov	Dec
18	99.38	99.90	97.99	100.00	135.32	165.52	102.20	103.79	100.40	150.66	246.96	104.76
19	99.44	99.90	97.99	100.00	135.36	165.49	102.22	103.76	100.35	150.67	246.96	104.87
20	99.45	99.90	97.99	100.00	135.36	165.53	102.22	103.76	100.43	150.67	246.95	104.87
21	99.44	99.95	98.51	100.00	135.58	165.52	102.28	103.76	101.62	150.65	246.97	105.18
22	99.38	99.82	101.30	100.00	135.31	164.09	102.24	103.79	100.36	150.67	246.87	106.24
23	100.87	100.04	99.94	100.26	136.33	150.57	101.57	103.79	99.69	148.89	251.40	107.76
24	101.05	100.04	99.92	100.27	136.16	151.21	101.59	103.79	99.72	148.81	251.54	107.69
25	100.87	100.14	99.94	100.26	136.32	151.00	101.62	103.79	99.71	148.89	251.40	107.73
26	101.21	100.02	100.10	99.95	140.85	141.66	107.29	103.07	100.13	154.12	249.87	103.96
27	101.32	100.06	100.06	99.98	141.34	153.66	107.30	102.96	100.28	153.89	249.60	103.48
28	101.29	100.06	100.07	99.98	141.42	153.61	107.64	102.96	100.24	153.89	249.66	103.50
29	101.30	100.06	100.06	99.98	141.68	153.64	107.75	102.97	100.24	153.84	249.65	103.55
30	101.37	100.06	100.19	99.99	141.37	153.20	107.55	102.95	100.28	154.02	249.62	103.54
31	101.19	100.02	100.24	99.99	141.75	153.58	107.66	102.96	100.30	153.95	249.60	103.16
32	101.26	100.14	100.00	100.00	141.58	153.99	111.48	102.96	100.23	153.63	249.69	102.06
33	101.96	100.14	100.00	100.01	141.74	154.10	111.51	102.96	100.24	153.65	249.69	102.06
34	103.76	100.35	102.73	100.37	144.91	162.29	106.26	102.56	100.42	157.08	255.90	102.07
