# Supplementary material for: A multi-objective mathematical model of a water management problem with environmental impacts: An application in an irrigation project
Source: PLoS One. 2021 Aug 3;16(8):e0255441. doi: 10.1371/journal.pone.0255441 (PMC8330924; doi:10.1371/journal.pone.0255441)
Supplement: Attachment — Submitted filename: Response to Reviewers..docx [file pone.0255441.s006.docx]

Dear Editor and Reviewers,

We would like to thank you for the careful review and thoughtful feedback on our manuscript “A multi-objective mathematical model of a water management problem with environmental impacts: An application in an irrigation project” (PONE-D-21-08160) and the opportunity to resubmit a revised copy.  We have revised the manuscript according to the comments and believe that it is substantially improved by incorporating these edits.

Below, we provide a point-by-point reply to the reviewers’ comments. We have included a marked copy of the revised manuscript that highlights changes and a clean version.

We appreciate your consideration of our revised manuscript.

**Journal Requirements:**

Please review your reference list to ensure that it is complete and correct. If you have cited papers that have been retracted, please include the rationale for doing so in the manuscript text, or remove these references and replace them with relevant current references. Any changes to the reference list should be mentioned in the rebuttal letter that accompanies your revised manuscript. If you need to cite a retracted article, indicate the article’s retracted status in the References list and also include a citation and full reference for the retraction notice.

**Response:** We have reviewed our reference list as per the PLOS ONE journal requirements in the “Vancouver” style.

**Additional Editor Comments:**

That paper has been largely improved after revision. However, the references need to be consistent. The authors are encouraged to use a referencing style in line with the Journal.

**Response:** We have reviewed our reference list as per the PLOS ONE journal requirements. Also, we have tried to be consistent in the references.

**REVIEWER COMMENT :**

**6. Review Comments to the Author:**

**Reviewer #1**: Although the authors already received many comments to improve the readability and presentation of the paper, the literature still needs to be normalized.

**Response:** We have checked the English sentences carefully and tried to improve the literature.
